# Adolescent Girls and Young Women Overcoming Adherence Challenges with Vaginal and Oral PrEP Use: A Longitudinal Qualitative Study from a Crossover Trial in South Africa, Uganda, and Zimbabwe

**DOI:** 10.1007/s10461-024-04503-y

**Published:** 2024-09-30

**Authors:** Mary Kate Shapley-Quinn, Siyanda Tenza, Destry Jensen, Thelma Tauya, Lydia Mampuru, Juliane Etima, Doreen Kemigisha, Millicent Atujuna, Lydia Soto-Torres, Sherri Johnson, Nombeko Mpongo, Nomsa Mhlanga, Kenneth Ngure, Ariane van der Straten

**Affiliations:** 1 Women’s Global Health Imperative, RTI International, Berkeley, CA USA; 2 Wits Reproductive Health and HIV Institute, Johannesburg, South Africa; 3 Division of Global Emergency Medicine, Brown University, Kigali, Rwanda; 4 University of Zimbabwe Clinical Trials Research Centre, Harare, Zimbabwe; 5 Makerere University-Johns Hopkins University Research Collaboration, Kampala, Uganda; 6 Desmond Tutu HIV Centre, Cape Town, South Africa; 7 Division of AIDS, National Institute of Allergy and Infectious Diseases, Bethesda, MD USA; 8 FHI 360, Durham, NC USA; 9 School of Public Health, Jomo Kenyatta University of Agriculture and Technology, Nairobi, Kenya; 10 Department of Global Health, University of Washington, Seattle, WA USA; 11 Center for AIDS Prevention Studies, Department of Medicine, University of California, San Francisco, CA USA; 12 ASTRA Consulting, Kensington, CA USA

**Keywords:** Adolescent girls and young women, HIV prevention, Pre-exposure prophylaxis, Adherence support, Sub-Saharan Africa, Qualitative

## Abstract

Rates of HIV acquisition remain high among adolescent girls and young women (AGYW) in sub-Saharan Africa. We explored South African, Ugandan, and Zimbabwean AGYW’s experiences in a crossover trial of two HIV prevention products: Daily oral pre-exposure prophylaxis pills and a monthly dapivirine vaginal ring. A subset of participants (n = 25) across all sites completed up to three serial in-depth interviews (SIDIs). The SIDIs explored barriers to product use, coping strategies, and the resulting outcomes. Coded textual data were analyzed using a product acceptability conceptual framework. Participants in the SIDIs described managing the array of challenges they encountered through formal adherence support, strategic product disclosure, and personally adapted strategies. For both products, perceived discreetness of the product and decision-making around disclosure was an important component of participants’ narratives. Participants tailored their coping strategies based on available personal resources (e.g., cell phone alarms for PrEP reminders, social support through disclosure) or study provided resources (e.g., encouragement from staff, adherence groups). Notably, challenges participants encountered with each product during the crossover period helped inform product selection during the choice period. Our findings suggest that—even in a context where AGYW have access to several options for HIV prevention—challenges to consistent product use remain, but accessible support mechanisms and informed choice can help mitigate these challenges. Enacting that choice may also empower AGYW to reach their short and long-term life goals—including for HIV prevention. (NCT03593655, 20th July 2018).

## Introduction

Global rollout of oral HIV pre-exposure prophylaxis (“oral PrEP”) is an important step towards providing effective and accessible HIV prevention options. However, adherence remains a challenge among adolescent girls and young women (AGYW) in sub-Saharan Africa (SSA), where they have three times more new HIV infections than their male counterparts [1]. Stigma towards PrEP users, rumors about PrEP use, male partner relationship dynamics, the burden of daily dosing, and beliefs related to efficacy have all been reported as challenges with using oral PrEP [2, 3]. Among those who do initiate oral PrEP, there are significant social, cultural, and behavioral challenges with executed adherence [3]. Advocates and researchers have pushed for increased attention and resource investment in additional HIV prevention strategies that will offer end-users the choice to use a product that fits well with their social context, personal preferences, sexual behaviors, and life stages [4–6]. While daily oral PrEP offers high efficacy (99% for sexual encounters) when taken as prescribed [7], the monthly vaginal ring (“vaginal ring” or “the ring”) has shown lower efficacy in Phase III clinical trials (approximately 30%) [8, 9], with higher efficacy (39–62%) using statistical modeling shown in the open label extension studies [10, 11]. However, the challenges that many users encounter with adhering to the daily regimen of oral PrEP (and thus reduced effectiveness) is potentially lightened with the vaginal ring, as it is meant to be replaced monthly once inserted [12]. Notably, recent data from early roll-out efforts in Zimbabwe show higher persistence among those who chose the vaginal ring, and comparable seroconversion rates between those who chose the vaginal ring and those who chose oral PrEP [13].

With the vaginal ring now prequalified by the World Health Organization (WHO), included in the WHO’s clinical recommendations for HIV prevention and approved or undergoing regulatory review in several SSA countries [14], it is important to build understanding of the users’ experience with additional PrEP options and the key strategies that can be implemented to support users in successful HIV prevention product use. Previous research has described users’ experiences with each of these products individually [15–18]. However, there is a gap in understanding how users’ experiences unfold longitudinally—with the possibility of choosing and/or switching between active products.

During the MTN-034/REACH study (a randomized crossover trial focused on “using and choosing”), we invited a subset of participants to complete serial in-depth interviews at three different time points. This longitudinal analysis sought to explore the challenges encountered—and corresponding management strategies that AGYW employed—with these oral PrEP and the vaginal ring. These challenges and strategies were analyzed throughout the study while participants used each product for approximately six months and following their choice of which study product (if any) to continue using during the last six months of the trial.

## Methods

### Study Design

**Fig. 1:**
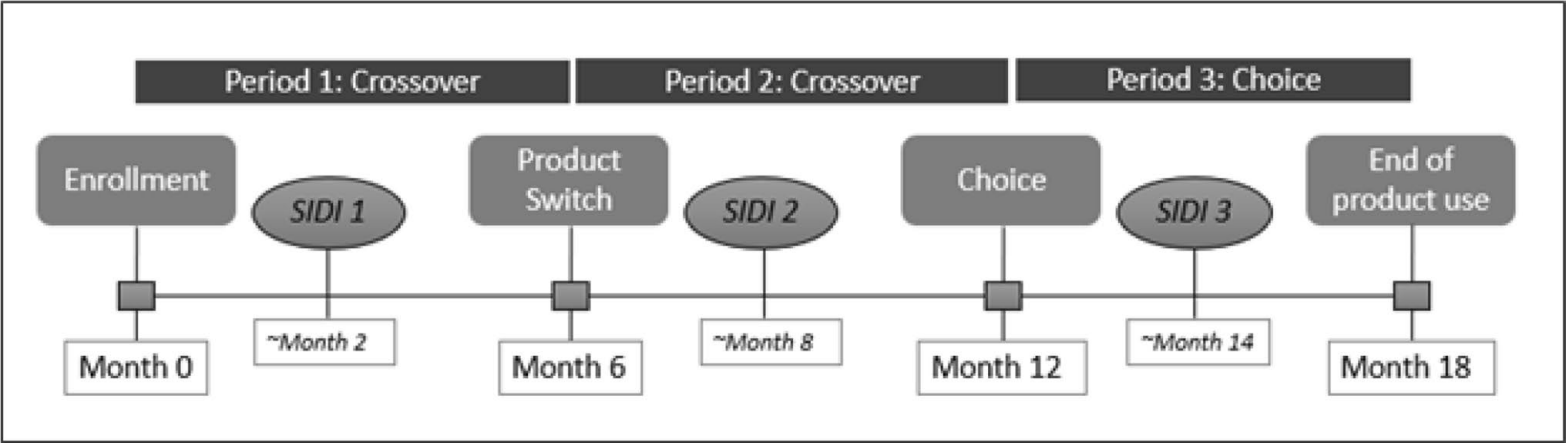
Schematic of study periods and serial in-depth interviews. The first interview (SIDI1) was focused on understanding the experiences early in the study, and initial experiences with the first product to which they were assigned. The second interview (SIDI2) similarly asked questions about initial experiences with the second product to which they were assigned, and also asked them to reflect back on their first product, comparing experiences with each. Finally, the third interview (SIDI3) delved into how the participant thought about the options (vaginal ring, oral PrEP, or neither), considerations when making their choice, and experiences to date with their chosen product

MTN-034/REACH (hereafter referred to as REACH) was a Phase 2a, randomized, open-label, crossover study conducted at four research sites in three countries: Zimbabwe (Harare), South Africa (Johannesburg, Cape Town), and Uganda (Kampala) between January 2019 and September 2021 (https://clinicaltrials.gov/study/NCT03593655) [19]. The primary objectives of the study were to collect safety and adherence data for two PrEP products—the monthly dapivirine vaginal ring (“vaginal ring” or “the ring”) and daily oral emtricitabine/tenofovir disoproxil fumarate (FTC/TDF) pills (“oral PrEP”) in AGYW, and to assess acceptability and preference for the products. The trial enrolled 247 HIV-uninfected AGYW between the ages of 16–21 years old. The design, procedures, and primary findings have been previously reported [19]. Briefly, this was an 18-month crossover study of the vaginal ring and oral PrEP, in which participants were asked to “use and then choose.” Participants were randomized to one of two sequences of the ring or oral PrEP and used each for a period of six months (“crossover period,” see Fig. 1). At the 12-month visit, participants were given the option to choose oral PrEP, the vaginal ring, or neither product for the subsequent six months (“choice period,” see Fig. 1). During this period, participants could switch between options after making their initial choice. Throughout the study, adherence was measured by assessing tenofovir diphosphate levels in dried blood spots after periods of oral PrEP use, and the residual amounts of dapivirine in returned vaginal rings after periods of vaginal ring use (further details reported elsewhere [19]).

### Longitudinal Qualitative Procedures

Serial in-depth interviews (SIDIs) were conducted with a stratified random subset of REACH study participants (n = 25 total, approximately six participants per site) to assess longitudinal changes in participants’ personal life, study participation, attitudes about and experience with each product. Specifically, SIDI guides were informed by a conceptual model of product acceptability related to adherence published by Mensch et al. [20] and designed to help understand factors that impacted and challenged product use, how participants coped with challenges, and how HIV prevention fit into the participant’s broader life context. SIDIs were designed to occur once during each study period (first assigned product, second assigned product, and choice period), approximately two months after the beginning of each period (see Fig. 1) to explore how participant experiences (and their descriptions thereof) shifted over time.

All REACH participants were stratified by study sequence, geographic location (study site), and age to allow for balanced representation of experience across these categories. From those strata, a sub-set of participants were randomly selected for the qualitative component, and consecutively invited to the SIDI activity, until one participant per sequence accepted from each site and age group (16–17 years, 18–19 years, and 20–21 years). All interviews were conducted by study-trained and experienced in-country qualitative interviewers, and lasted an average of 62 min. During the time when COVID-19 related restrictions were in place, interviews were still conducted in person with social distancing, masks, and sanitizing protocols in place. All SIDIs were audio recorded and transcribed, with single-step translation used if portions or all the interview was conducted in a language other than English. All transcripts underwent a standard quality control process. Full procedures for data review are available [21].

### Analysis

A codebook was developed through inductive (i.e., review of interview summaries) and deductive (i.e., research objectives, theoretical frameworks, and codebooks developed for previous similar studies) processes, by members from the qualitative data management center, the management team, and research sites. The codebook was also informed by two analytical frameworks of relevance to REACH: The previously mentioned conceptual model of product acceptability by Mensch et al. and the Psychological Empowerment Framework [20, 22]. The latter framework was included to inform a separate research question and was not applied to the current analysis. The codebook was iteratively refined through extensive discussion and testing application of the codes to study transcripts.

After codebook finalization, all transcripts were coded using a web-based qualitative coding software (Dedoose Version 9.0.17, SocioCultural Research Consultants, USA, Dedoose.com), by a team of six analysts. Weekly meetings included all coders, representatives from each site who reviewed coded transcripts for consistency and completeness, and protocol team members. Inter-coder consistency was assessed through tests using the training tool in Dedoose, and weekly reviews of coded transcripts by a designated reviewer. Any disagreements or discrepancies were discussed, consensus was reached, and updates to coded transcripts and/or the codebook were made, as necessary.

To analyze longitudinal factors related to challenges and facilitators to product use during the three study periods, code reports that aligned with four topics derived from the Mensch conceptual model of acceptability were identified: (1) Individual level factors (example associated codes: *Developmental growth, Mental health*), (2) Social/contextual factors (example codes: *Family, Organizational influencers, Partner)*, (3) Product use experience & acceptability (example codes: *Effect on life, Initiation, Execution/compliance)*, and (4) Product choice and preference (example codes: *Choice, Comparison/preference*). For each SIDI participant, analysts wrote summary memos of the four identified topic areas and mapped these across each timepoint where a participant was interviewed. The analyst team reviewed the analytical matrix for time trends and themes within participants. Co-authors at each site reviewed a selection of SIDI summaries to achieve alignment with themes and trends identified.

While analyzing the code reports aligned with the product-centric Mensch conceptual model of acceptability (i.e.: Individual factors, social/contextual factors, product use experiences & acceptability, product choice/preference), important themes emerged across these code reports that aligned with a more “user-centric” view of the data, rather than a product-centric one. The analysis team decided to shift the framing of the findings away from the Mensch conceptual model, and towards the below-presented themes that emerged from the analysis of code reports. These emerging themes were well suited to capture the longitudinal nature of the dataset.

For case studies, the analysis team worked together to identify specific participants that represented each study site, and who had a collection of experiences that aligned with the emergent themes. Co-authors and analysis team members from each site closely read, edited, and added details to that site’s case study (as appropriate) to ensure accurate representation of the participant’s experience.

## Results

The REACH qualitative longitudinal sample included 25 participants who, by design, were evenly distributed across three age subgroups (16–17, 18–19, 20–21) and geographic locations in which the REACH study was conducted. Table 1 shows basic demographics of the serial in-depth interview (SIDI) sample compared to those of the overall REACH study population. Three of the participants in the longitudinal sample did not complete all three interviews: one participant only completed her first interview before moving out of country, one participant missed her second SIDI due to COVID-related lockdowns, and the third participant never completed her third SIDI due to relocation to another country.

**Table 1:** Longitudinal participant demographics at enrollment, compared to overall sample

	SIDI		Total	
	N	%	N	%
Total	25	(100)	247	(100)
Age—mean, median, interquartile range	18.1, 18	(17–19)	18.4, 18	(17–20)
Currently in school	10	(40)	92	(37)
Secondary education completed	22	(88)	212	(87)
Earns income	6	(24)	53	(22)
Parous	11	(44)	99	(40)
Any vaginal sex in past 3 months	20	(80)	203	(82)
Number of sex partners in past 3 months				
None	2	(8)	12	(5)
One	15	(60)	145	(59)
Two or more	8	(32)	90	(36)
In partnership for at least one year	15	(65)	133	(61)
Married traditionally* or cohabitating	5	(20)	30	(12)
Received goods or money for sex in past 6 months	4	(16)	66	(27)

^*^No participants were legally married

Across SIDI participants (and similar to the full REACH sample), product adherence was generally high during each study period, across age groups, and locations. As shown in the heat map in Table 2, the majority of cells are green and yellow, indicating months where adherence data was received and showed moderate or high adherence for each participant in the longitudinal qualitative sample. Two participants (one in each sequence group) received adherence results during the first product use period showing low (or no) adherence to the product during that month, and it is worth noting that each of those participants chose the alternate product (for which they had not received “red” results) in the choice period.

**Table 2:**
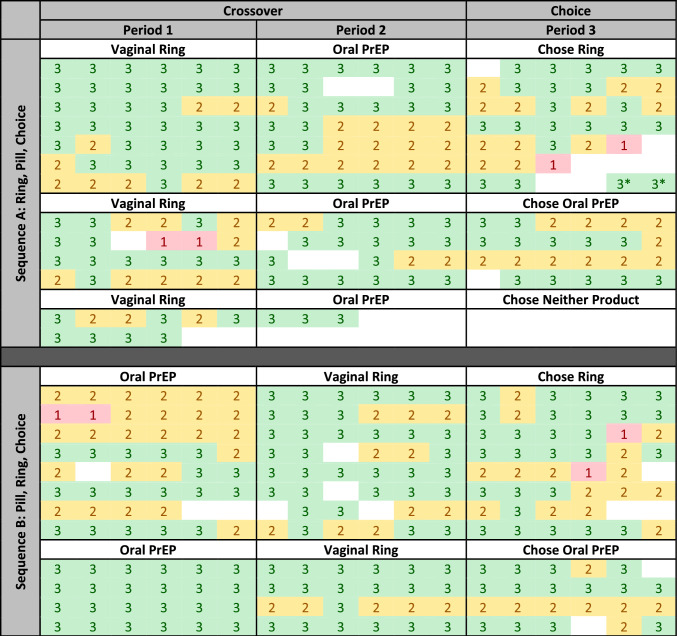
Heat map of adherence to study product throughout period of participation, by sequence, for all 25 participants who participated in serial in-depth interviews

Each row represents a single participant who was in the Serial IDI sub-sample. Each cell in the row represents one month in the study (a total of 18 cells, divided into 3 sections to align with each study period)

Color coding is indicative of level of adherence to study product. These adherence levels were assessed monthly using dried blood spots (while using oral PrEP) or residual dapivirine in used vaginal rings.

Indicates high adherence

Indicates some to moderate adherence

Indicates Low adherence/ non-use of product.

Blank cells indicate months where no adherence data was collected, due to missing the procedure, or early departure from the study.

*Switched from vaginal ring to oral PrEP in Period 3

SIDI participants described challenges to using each of the HIV prevention products (as assigned during the crossover period) or chosen (during the choice period), and coping mechanisms to overcome these. Participants’ narratives over time showed that product-specific experiences shifted, including around product disclosure (and/or study involvement), social support for product use, side effects, and how products impacted day-to-day life. Concurrently, as their participation in the study progressed, AGYW reported evolving relationships in their family and social support networks, drastic disruptions due to the COVID-19 pandemic, and evolving personal circumstances. Together, these elements impacted how participants thought about HIV risk, prevention, and overall personal health. For about a third of participants, the strategies they employed to overcome challenges to study product use were explicitly linked to their desires to stay HIV-free, and to maintain their overall health. Table 3 outlines examples of how each of these topics (further described in the results below) emerged out of code reports based in the Mensch conceptual model of acceptability.

**Table 3:** Examples of Mensch conceptual model of acceptability integration with emerging themes

	Code report topics			
Emergent themes	Individual level factors	Social/ contextual level factors	Product use experience	Product choice & preference
Challenges to product use		Discreetness and inadvertent disclosure, rumors, COVID-19	Side effects (nausea, vaginal discharge)	
Strategies to overcome challenges to product use	Linking motivation to adhere with HIV prevention goals	Study-provided adherence support	Drinking more water with oral PrEP to help swallow	Using choice as a way to avoid least-tolerable challenges
Achieving desired HIV protective behaviors and overall health	Progress and setbacks for education/ financial goals		Using HIV prevention products and feeling “safe”	Product choice as an element of feeling empowered

### Challenges to Product Use

Participants discussed challenges with oral PrEP use (including side effects) more prominently than ring-related use challenges. In contrast, the discussion of anticipated issues with oral PrEP were minimal—as opposed to the ring.

Side effects that participants often associated with oral PrEP included drowsiness, dizziness, nausea, diarrhea, vomiting, changes in appetite, and headaches. Additional challenges that were dominant among participants while using oral PrEP included the large size of the pill making it hard to swallow, unpleasant taste, and the burden of remembering to dose daily.

Though less prominent, participants also described several challenges they experienced with ring use, although anticipatory concerns dominated compared to actual experiences of problems they encountered. Most frequently mentioned among these were anticipatory concerns that a male partner would feel the vaginal ring during sex or that the ring may fall out during day-to-day activities. Actual experiences centered on feeling discomfort with the ring in place during daily life, the ring coming out during sex, dislike of having a foreign device inserted in the body, and associated side effects such as vaginal odor, itchiness, and vaginal discharge.

The perceived discreetness of each product—including intended and inadvertent disclosure—influenced participants’ preferences and specifically bridged key product attributes to the inherently social aspect of disclosure of PrEP. Concerns around inadvertent disclosure of the ring centered around it falling out or being felt during sex. For oral PrEP, participants were concerned about accidental disclosure through someone discovering the pill bottles, hearing pills rattling in their bottle, or being seen taking their pills.

Themes of discreetness as it relates to product disclosure were woven throughout the different social challenges participants mentioned. Participants often initially experienced difficult reactions from friends, family, and partners when disclosing product use. Participants commonly reported encountering reactions of shock, anger, or misunderstanding after first disclosing product use, though they also mentioned that—more often than not—those reactions turned to relationships of support over time. One participant described an initial challenge with her mother: *“…Mummy didn’t know so when she got to know she got annoyed saying ‘how can you do something when I don’t know?’… She didn’t know because I didn’t want them [study staff] to tell her and even without her knowledge, I would take the pills but eventually she saw them. So, when she saw them I told her they have no problem…, she got annoyed a bit but after calmed down… Actually she reminds me”* (Kampala, age at enrollment: 17 yr., SIDI #1). Unique to periods of ring use were instances where sexual partners’ discovery of the participant’s ring occurred when it was felt during sex, inevitably opening conversations about product use at a challenging moment. In this longitudinal sample, though disclosure raised difficult discussions with sexual partners, no participants reported violence from sexual partners due to disclosure of use of either study product.

Other social challenges such as social stigma, and community rumors were not directly tied to disclosure, but nevertheless impacted participants’ experiences with product use. Rumors in the community were particularly notable as reported by participants at the Harare site, with one participant saying, *“Some say that ‘It’s for the Satanists’, or that wearing them causes cancer or that when you want a baby you won’t be able to conceive, so it has side effects”* (Harare, 18 yr., SIDI #1). The misinformation and stories circulating in communities varied widely between different study sites and included claims such as study participants being satanists or prostitutes, study staff giving participants HIV and selling their blood to foreigners, study products causing mental health issues, and product use causing infertility. One participant enrolled at the Johannesburg site was assigned to the vaginal ring for her first period of product use. When asked what community members said about the ring, she responded, *“They were asking me if I am not scared that when I use the ring it will stop me from becoming pregnant, that the ring will make me infertile”* (Johannesburg, 17 yr., SIDI #1).

These community-level stories, in some more extreme cases, were taken up by members in a participant’s social circle, which then pressured a participant to stop product use. One participant was supported by a friend, her husband, and heard encouraging information about oral PrEP from others in her community. However, one conversation with women in her neighborhood caused her to stop taking the pills for over 2 weeks. She said, *“They told me that ‘That medicine can infect someone with HIV, it has HIV.’ And then I was like ‘Why would they give us medicine that has HIV, do they want us to get infected?!’ and they said, ‘How do you think they benefit from helping you not get infected with HIV.’ And they continued with so many words and I got scared and I was like ‘I am not going to take it [pill] again.’… I spent sixteen days not taking it. …I asked them [women in the neighborhood] ‘Now, for this medicine that I have not taken, can I take it back?’ and they said ‘No, do not take it back’ and they told me to throw them, and I threw all the sixteen pills. I threw all of them and wondered ‘Now about those that I have taken, haven’t I already got infected?’”* (Kampala, 18yrs., SIDI #1).

Participants also described contextual and structural factors that made using study product challenging, particularly for oral PrEP. Those who were attending school or working jobs often found that their schedules conflicted with their planned time to take their pills. Even in cases where the scheduling was not normally challenging, when the schedule changed or unanticipated events arose, these participants encountered adherence issues. This was particularly true for participants who wanted to conceal oral PrEP use from others, as they were careful about where they kept their pills and who might see them when taking the pill.

The advent of COVID partway through the study drastically impacted participants’ access to study products, and the context of use. The Cape Town site commenced their first SIDIs (SIDI1s) in the latter half of 2019 (later than all three other sites) with some SIDI1s, most SIDI2s and all SIDI3s all occurring after March 2020. So, measures to manage COVID-19 occurred before or during the SIDI sequence for all of these participants. Given this timeline, discussions of COVID-19 measures were particularly notable in the Cape Town interviews. Some participants were dispensed more than one ring at a time to reduce the number of clinic visits needed, introducing the element of at-home storage of vaginal rings and the discreetness-related concerns that ring storage may raise. Participants taking oral PrEP similarly had additional pill bottles to store. When local and national governments implemented strict lockdowns, this impacted work and schooling schedules, thereby impacting pill-taking routines. One participant in Cape Town recounted that COVID-19 precautions impacted her working hours and caused her to miss her scheduled time for taking oral PrEP. She said, *“The hours that we worked at my work were not as normal as they were before the lockdown. So, sometimes; normally I took my pills when I came back [from work] … sometimes we would work until the time passes and you would find that the time, I am supposed to take the pills; I would still be at work because the lines were long.”* (Cape Town, 18 yrs., SIDI #3). If not already living with a male partner, lockdowns also changed the context of dating and sexual relationships that shifted the need for disclosure, opportunities for inadvertent disclosure, and the context of risk as it relates to sexual encounters.

### Strategies to Overcome Challenges to Product Use

Throughout the three longitudinal SIDIs, participants discussed how they managed product-related challenges, and how that may impact their future behaviors around product use. Strategies to cope with these challenges included modifying individual behavior, implementing social strategies, and accessing resources drawn on through the study clinical environment.

#### Modifying Individual Behavior

For some product-related challenges like swallowing pills on a daily basis, and the novelty of having a ring placed in the vagina, participants mentioned the passage of time (and perhaps, by association, gaining experience with a product) as a key factor in *“getting used to it”* (Cape Town, 20 yrs., SIDI #2). Participants also described simply choosing to ignore the rumors, stories, and misinformation they heard from their social circle about the study products. For overcoming the other challenges described above, some participants described anchoring themselves in a sense of purpose they had identified such as their personal goals for HIV prevention, staying healthy, or longer-term life goals. One participant in particular described feeling that she had a “choice” to stay HIV-free since she was not born with HIV which contrasted with some family members who were born with the virus, and thus a sense of duty to protect herself: “…*It was right for me to prevent [HIV] since I was not born with it. So I have a choice not to have HIV*” (Cape Town, 17 yrs., SIDI #1). Participants also described family members and friends living with HIV as sources of motivation for overcoming the challenges they faced with product use, so they could maintain their current HIV negative status.

However, many participants found that overcoming the challenges they identified required deliberate proactive actions. For participants who faced difficulty with taking oral PrEP, varied and sometimes creative strategies were used to help mitigate these issues (summarized in Table 4).

**Table 4:** Summary of individual behaviors implemented to overcome challenges with taking oral PrEP

Oral PrEP challenge	Individual behaviors as strategies
Challenge taking pill at target time every day	- Refine target time to fit with work/school schedule - Implement system of alarms/reminders from others/timing alongside regular activity, like a TV show - Allow for flexible timing when taking the pills
Experiencing side effects when taking oral PrEP	- Drink extra water - Sequence dose with food - Change time of day dose taken
Trouble swallowing oral PrEP	- Drink more water with dose - Crush pills and dissolve in water^a^ - Break pills in half^a^

^a^Note that crushing pills or breaking them in half were neither recommended nor condoned by the study team or the product and study sponsors. These were strategies that participants devised on their own to address the challenges they encountered, accentuating the need to have acceptable ways to accommodate pill-taking that are also aligned with dosing recommendations

Notably, some participants mentioned how they tailored their product choice in the third period of the study based on the challenges they had faced during the crossover period when they were randomized to each product, effectively allowing the participants to “choose the challenge(s)” they found more feasible to manage. One participant, for example, did not like the size or the material of the ring, but ultimately chose that product because she found the dosing regimen and the side effects of the pill to be more challenging. In her SIDI #2, she said that she disliked the size and material of the ring, and would have preferred a tampon shape, with a cotton or sponge texture. However, in her SIDI #3, she reflected on minor challenges with both products (yellowish discharge, lower abdominal pain with the ring, aftertaste, and side effects with oral PrEP), and thought both were relatively easy to use. She said she got used to the dosing schedule of oral PrEP and found the vaginal ring comfortable. Ultimately, she said*, “I had thought of choosing [oral PrEP], but I didn’t want to find myself forgetting to take it, so I thought it will be better to choose the ring… I choose the ring because PrEP made me sick”* (Johannesburg, 20 yrs., SIDI #3). Another participant mentioned her fears around being labeled as having HIV, so felt she could not disclose her use of oral PrEP. To avoid this challenge around disclosure and fear of stigma, she also chose the ring in the last period of the study.

#### Implementing Social Strategies

A critical component of successfully coping with many challenges was the social support participants received. Inevitably, for participants to gain social support that would mitigate or buffer against challenges (such as adhering to dosing regimen, rumors and gossip about HIV prevention products, judgmental comments from those in their social circle), some degree of disclosure about product use was necessary. The decision-making processes around disclosure of product use were varied and tailored to each participant’s particular situation (summarized in Table 5**)**. Importantly, participants described differing levels of willingness and readiness to manage challenging reactions to disclosure, so situations that felt “manageable” to one participant may not have felt manageable to others.

**Table 5:** Summary of social strategies implemented to overcome challenges with taking oral PrEP or using ring

Social challenge	Social strategy
Unsure about reaction if voluntarily disclosed product use	- Observe reactions when others disclosed product use - Just disclose one product (often oral PrEP) to “test” reaction first
Worry that ring may be felt during sex (causing relationship strain, accusation of promiscuity, mistrust)	- First disclose only one product, or different product (like contraceptive) to “test” reaction - Disguise as other product. “*I have never felt it [the ring]. But there is someone I had sex with and he asked about the family planning method I was using. I told him that I was using the LOOP. He said, ‘Go to the clinic to have it checked if it is well placed because I felt like being prodded.’ Then I just said, ‘Its ok.’”* (Harare, 20 yrs., SIDI #2)
Male partner resistance/displeasure after disclosure	- Terminate relationship - Continue product use without partner’s knowledge

Despite the challenges participants faced around disclosure itself, they employed many strategies to manage the situations, which allow for sharing about their product use when they wished, and how they wished. For those who were able to do so successfully, they spoke of the benefits of having support from their male partners, family members, and others in their social circle, thereby enabling practices that allowed for good adherence.

#### Accessing Resources through the Study Environment

Participants described strategies to manage product use challenges that were based on the clinic or study environment. Many successful strategies for coping with product-related side effects were provided by study staff, such as education about HIV prevention and expected side effects, encouragement to persevere and let side effects pass, treatment of side effects attributed to product use, and adherence support intervention that included a menu of options like support groups (virtual or face-to-face), counseling, and reminders. This took the form of moral support or encouragement to persist with product use until side effects dissipated, managing expectations around side effects to normalize them, or conversations with clinic staff about rumors and social challenges that participants sought assistance in overcoming.

Participants also interacted with each other either casually in the clinic waiting room, or in study-organized events such as study parties and adherence clubs. In these peer interactions, participants mentioned learning others’ strategies to improve adherence (like setting a phone alarm or keeping her pill bottle near her bed) and how others were experiencing and managing any side effects. Participants were also able to glean each other’s experiences with disclosures specific to the study context and take those into consideration to tailor their own disclosure decision-making processes.

### Achieving Desired HIV Protective Behaviors and Overall Health

Through the format of serial in-depth interviews, participants had opportunities to share their aspirations and goals for their lives with the interviewers and reflect on the evolution of those goals over the course of (up to) three interviews. In discussing their hopes and motivations upon joining REACH, participants talked about reasons closely tied to the study offerings (contraception, HIV prevention), as well as goals of financial independence, further education, and wishes to provide support and housing for family members. While some participants were able to make progress towards their financial and educational goals during REACH participation, more participants experienced setbacks due to COVID restrictions that interrupted workplaces and schooling. Nevertheless, participants who were able to successfully employ strategies to overcome barriers to study product use also reported feeling confident in their ability to eventually achieve their personal goals. This was verbalized in different ways: some spoke just of feeling “protected” or “safe,” others mentioned HIV prevention as helping to feel freer in enjoying sex, and others discussed being able to feel happier and more confident in their ability to achieve their longer-term life goals and aspirations.

One participant spoke enthusiastically about going to university during her first interview, and tied her REACH participation to wanting HIV prevention methods for when she went to university (associating that with parties and new partners). By her third interview, she had limited involvement with schooling due to COVID restrictions, was worried about upcoming finals, and her household income had diminished because of decreased work opportunities. Still, she was motivated to adhere to the study product and linked it to her future, saying, *“I told myself that I love myself and if I want to live better and a healthy life I have to go on with the study”* (Johannesburg, 17 yrs., SIDI #1).

In sum, participants felt that learning about and successfully implementing HIV prevention through their study participation empowered them to make their own decisions to protect themselves. For those who were able to confront and mitigate their barriers to product use, those choices and decisions led them to feel protected, and aligned with continued hope for bigger life goals.

### Case Studies

The below case studies were selected to help show, concretely, how the above-described themes were highlighted by participants. These cases were selected to demonstrate the varied geographic and contextual environments that participants joined from, and we attempted to select cases where a single participant demonstrated many of the above themes across three different interviews.

Cape Town Experience (17 years old at enrollment) The participant was using oral PrEP during her first study period, and was a bit worried about the prospect of switching to the ring, but was otherwise happy with the study and use of the pills. This participant’s mother also was using oral PrEP. Others she knew used oral PrEP to varying degrees. Her boyfriend knew about the study and study products and was supportive of her participation. She had some initial concerns about what it would be like to swallow the oral PrEP pills but found them easy to swallow. She had occasional instances where she would have trouble taking her daily dose as intended, sometimes feeling “lazy” to take the pill on a given day, but overall had good reminder systems in place to support adherence to daily pill taking.

In her second interview, the participant shared how COVID-19 lockdown measures had changed her life. She switched to using the ring and told her parents, sister, and one friend, but did not want to tell others as she was afraid of being judged and she was not sure how they would react. Her sister and friend’s reactions were shock at first, but the participant seemed to expect this reaction, saying: *“everyone when it is said it is something you will insert in the vagina, they have the fear that okay; they get shocked.” *She was scared and stunned when she first saw the ring herself and thought it would be painful to use. However, since she inserted it, she did not have a problem. She explained, "*I even forget that it is inside me. I walk okay and I am comfortable*.” She was given a second ring because of limited clinic access during lockdown, was unsure if she would be able to change it on her own, was able to do it and then felt comfortable changing the ring on her own. Though the participant initially thought she would prefer oral PrEP pills, she favored the ring in her SIDI2 because she was comfortable with insertion and found the product painless. She also found herself forgetting to take the pills at times, which influenced her preference for the ring.

In her third interview, the participant described still spending most of her time with family and friends but still feeling the impacts of COVID-19. Her long-term goals included studying accounting at university, and she saw dating and peer pressure as a potential threat to her goals. She mentioned she did not have a boyfriend, though she was sexually active. When she discussed how she might describe the study products to others, she included messaging about potential side effects, but also highlighted that women who use the products could “*feel free*” and know they are protected. She ultimately chose the ring, saying “*If I have inserted the ring, I am 100% sure that it will protect me more than the pills that I usually forgot. It is in my system*.”

Harare Experience (20 years old at enrollment) In her first interview, the participant talked about being assigned the ring and initially communicating with others in her community about her product use. However, she described later feeling as though it was easiest to only talk to those already in the study about her ring use, saying that when she talked to people outside the study about ring use, “*you will end up making explanations... I noticed that it can distract me from using the product or even to influence me to leave it.*" She discussed proudly showing public support for the study and overcoming challenges to be able to attend study support groups. She found that she didn’t notice the ring at all while it was in place and had no trouble leaving it in place. While she had initial concerns about removing and replacing the ring during menses and diseases she may contract from ring use, she noted that she had not experienced such after two months of product use.

In her second interview, the participant described requesting additional support from the study to help her adhere to the daily dosing schedule for oral PrEP, anticipating that she would have challenges with adherence. She continued to describe herself as a study champion – often talking about the study to others and taking her pill in front of others. She reported stomach pain and diarrhea soon after starting oral PrEP but was able to come to the study site and was given medication that resolved these issues. She said she initially was wary of the large size of the pill, but quickly got used to it and felt like she could adhere well.

In her final (third) interview, the participant talked about wanting to continue preventing HIV *“…so that if I get a job or go to school, I will go there without disturbances rather than being a sick person. Sometimes you wake up not feeling well and you won’t be able to go to work or to work for your family."* Lockdowns related to COVID-19 pandemic occurred between her second and third interviews, so she discussed using WhatsApp groups to stay in touch with young women in her community and share how they were managing during the lockdown.

While she experienced challenges with daily dosing and side effects with oral PrEP, she got used to them over time. With the ring, she was slightly uncomfortable with the increased vaginal fluids, but preferred that over the pill’s side effects. This participant found it easier to disclose use of oral PrEP than the vaginal ring, and she didn’t feel comfortable talking about the ring with her father and brother – anticipating lots of questions. Ultimately, the participant ended up choosing the ring in period 3, saying she felt "*self-assured as someone who is protected*".

Kampala Experience (18 years old at enrollment) Throughout all three interviews, this participant discussed having the same primary male partner who she lived with, and was financially supported by. She joined the study as she suspected her partner had other partners, putting her at risk for HIV. She was also inspired by the staff she met through recruitment, saying, *“you never know in future I could also be like them where I could help people, researching about medicine that prevents infections.”* When she first joined the study, she had two additional male partners who provided her financial support, though she had separated from these partners by the time of her first interview to reduce HIV risk, telling the interviewer that she greatly fears HIV as she knew it had no cure and she had family members who had HIV and passed away. Though she experienced side effects that she effectively managed when she started taking oral PrEP, she stopped using the pills for over 2 weeks after hearing from women in the community that the study tablets were intended to spread HIV. As part of the adherence support clubs in the REACH study, two youths living with HIV visited with study participants. This visit was extremely influential for the participant, and along with encouragement from study staff, she resumed use of oral PrEP (though in her next interview, reported she still forgot them occasionally).

During her second interview, the participant reported feeling more protected using the ring (compared to oral PrEP) since it is always inserted. At first, she said it looked big and thought it would cause pain, but she was surprised when she felt nothing once it was inserted, and she did not experience any side effects. Although she didn’t tell her partner she had switched to using the ring, he had known it was part of the study and asked her if she was using it after feeling it during sex. He said it caused pain, asking her to remove it for sex, and to re-insert it afterwards. The participant discussed this during an adherence meeting after being encouraged by other participants sharing freely, and study staff were able to bring her partner in to the clinic to talk to him, and after that, he encouraged her to always keep the ring inserted.

Shortly before her third interview, COVID restrictions had gone into place in Kampala. The participant spoke about her partner having reduced work because of COVID and reducing frequency of meals due to the lack of money, instead relying on friends and her partner’s mother for financial support. While she had spoken about interest in being involved with health research or being an ambassador for HIV prevention products in previous interviews, she did not mention that in this interview – the aspirations she spoke about were related to having a home with better living conditions, and avoiding any jobs that would require her to work at night. She chose the ring during the third period, saying that it reduced worry for her, and she was driven by choosing what would be easy: she liked that she cannot forget when it is time to replace it, and she felt free while using it (despite sharing that she still had some challenges during insertion). She also mentioned knowing that her adherence results would be good while using the ring, and admired others who were able to get good adherence results with oral PrEP (her drug feedback results were mostly “yellow”). She said, “*With the vaginal ring I am not worried at all, yet I would worry about the pill results because I would forget and miss taking sometimes.”* Her partner had also told her that he was supportive if she decided to choose the ring.

Johannesburg Experience (20 years old at enrollment) During this participant’s first interview, she appeared happy. However, when discussing others in her family, she brought up financial hardship in their household and the mood shifted. She felt good in her relationship with her partner and was working part-time. When she discussed the possibility of joining REACH, her mother shared that she (the mother) was living with HIV and encouraged the participant to join. She was randomized to the ring first and was happy with it, reporting no side effects and that her partner did not feel it during sex, and he was supportive of her using it. She was adhering well to the ring, saying, *“my results are geared on the green* [indicating high adherence, see Table 2]*, so I am very happy and proud of my results.”*

When she returned for her second interview, the participant again appeared happy at first, but her feelings about her relationship with her partner had changed. She felt he was unmotivated after losing his job and engaging in drug use. Her partner blamed her for his misfortune, and she complained of maintaining the relationship out of pity and fear he would harm himself if she left. She was concerned about COVID-19 and had stopped working due to COVID. Since her previous interview, she felt more confident about her health, saying *“I am very confident right now because I am fully aware of my health, and I know that in the REACH study… the study products that I get are very helpful towards my life and keeping me safe health wise and yes I am happy.”* She had switched to using oral PrEP and planned to choose it during period 3, despite some initial adherence challenges, saying *“At first, I wanted to go with the ring because it’s much easier to use and it is flexible to use, but then I thought again about the PrEP tablets. The PrEP you know protects against all modes of HIV transmission and the ring only prevents from sexual intercourse part.”* She forgot to take her pills in the morning due to a busy work schedule and switched to evenings, yet continued to miss doses despite an alarm and reminders from colleagues. The keychain holder for pills helped her remember as it can be in a visible place, and her mother also reminded her.

In her final interview, the participant described that her life was largely the same as in the second interview: Arguments and drinking at home and the stress in the relationship with partner seemed to be taking a toll on her. She had chosen oral PrEP, as she stated previously, partially due to the good adherence results she had received while she was randomized to the pills. It was still important to her that the pills provided systemic protection, citing her understanding that she could be exposed to HIV through her mother because they lived together. She had fallen into a routine of taking her pills at the same time every evening as a soap on TV ended, and at the same time as her mother took her ART. She described that her mother and partner continued to be supportive of her use of the study products. She planned to continue using oral PrEP after the study ended, saying *“I think staying HIV negative, I wouldn’t say it’s simple, no, it’s not simple but it’s doable if I take the necessary precautions. Once I finish my participation in the study, they will give me a referral to go and get my PrEP. I [will] most definitely go to the clinic and continue with it, continue taking the necessary precautions and using a condom all the time and go for testing so yes I am planning on staying negative for the rest of my life.”* She also discussed her desires and plans for the future: *“I have wasted 4 years of my life hoping to get a job… I want to go back to school so bad…. like I want to feel the pressure again of being a student. But at the same time, I am scared… the only reason why I am scared to go back to school is that I am scared of failing.”*

## Discussion

Serial in-depth interviews conducted in the multisite REACH study provided opportunities to understand relevant factors impacting HIV prevention product experience, including adherence, and choice. The inclusion of participants aged 16 and 17 ensured that perspectives of minors were well-represented, and the study design (use of each HIV prevention study product—vaginal ring and oral PrEP—for 6 months, then choosing which to use again or neither) allowed participants to make choices based on well-informed preferences learned from direct personal experience with both products. Three key findings were identified from the analysis of these longitudinal qualitative interviews. First, participants had the autonomy and systems of support needed to manage challenges encountered with use of both oral PrEP and the vaginal ring. Second, using each product prior to choosing allowed participants to factor their own ability to adhere into the choice they made. Finally, participants linked their successful use of HIV prevention products to feelings of increased optimism for their broader life goals.

### Autonomy and Support as Tools to Overcome Product Use Challenges

Multiple studies have shown that oral PrEP users in SSA regularly encounter challenges with taking it as prescribed, and PrEP persistence is low, especially among AGYW [23, 24]. Reasons that AGYW discontinue oral PrEP use as reported in previous research are varied, including low personal risk perception, dislike of the daily regimen, side effects, low social support, and stigma [3]. Though REACH is the first study to focus on vaginal ring for HIV prevention in AGYW in SSA, the women in pivotal ring Phase III trials and open-label extensions reported challenges related to vaginal rings being unfamiliar delivery mechanisms, concerns about sexual partner reactions, worry of use during menses, hygiene and the continuous presence of a device in the vagina [9, 15, 16]. Across participants in this qualitative sample, all these previously described challenges were encountered in some form. The support provided through the study context (adherence support, study staff) was instrumental in providing participants the motivation, ideas, and encouragement to explore which strategies would work best for them to overcome these challenges. Using the study-provided support and with the autonomy to explore their own ideas as well, REACH participants developed fine-tuned strategies over the course of the study to fit their specific life context, enabling them to overcome these challenges. These strategies included adherence support mechanisms provided through the study [25, 26], disclosure to others in their family and social circles [27], and strategies they came up with on their own as reported here (such as various reminder systems for oral PrEP doses, or the confidence to choose to ignore rumors). Further work is warranted to understand the role and impact of these different support mechanisms to guide implementation efforts in resource-limited settings. However, it is evident from this analysis and echoed in another analysis specifically of the formal study-provided adherence support [26] that offerings focusing on a strong rapport with counsellors and peers alongside frequent and flexible contact may be well-suited for the specific needs of AGYW interested in using HIV prevention methods.

### Using Before Choosing to Enable Adherence

A unique factor that contributed to participants’ ability to overcome challenges to product use was the “use and choose” study design in REACH (further explored in another analysis [28]). Experiencing each product, along with its benefits and challenges, allowed participants to understand what would be needed to be successful with each product. Thus, based on actual experience, participants were able to tailor their product choice to the one that best fit their personal preferences and unique life context.

The contraceptive field’s literature shows that offering more options improves reproductive health outcomes for women who want to prevent pregnancies, and better method coverage at a population level [29, 30]. Similarly, in HIV prevention, the anticipation is that providing more PrEP options will lead to better choice and enhanced protection for all people [4]. Results of the SEARCH Dynamic Choice study support this, showing that options for HIV prevention increased coverage greatly and reduced HIV incidence [31]. The addition of novel options like the Dapivirine vaginal ring and injections (e.g., long-acting cabotegravir and long-acting lenacapavir [32, 33]) to the HIV prevention product mix, thus, raises important questions around how to successfully support end-users in uptake of and adherence to these various HIV prevention methods when they become available. Recognizing that there is no “silver bullet” in HIV prevention, we can also anticipate that users will want to try more than one option to assess their fit, as they did in REACH and described in this paper, leading to switches between available products as their needs, preferences or circumstances change [34]. Trying and switching between products may be easier with fully user-controlled and reversible methods like condoms, oral PrEP and the ring, and operationally more challenging for options such as the long-acting cabotegravir injections which are not reversible once administered.

### From Overcoming HIV Prevention Challenges to Reaching for Bigger Life Goals

A key finding from the longitudinal interviews was that each product had its’ set of challenges, and overcoming product-related challenges during REACH was instrumental for many participants. Overcoming challenges to achieve better protection from HIV (via better adherence) was empowering, and that empowerment was linked to self-efficacy in other areas of life. Previous evidence shows that supporting decision-making around health in adolescence can promote feelings of agency and self-determination [35]. The narratives from these REACH participants also suggest that this confidence-building through informed product choice, problem-solving around challenges encountered, and participant-centered adherence support may have increased participants’ feelings of agency or self-efficacy with ripple effects across participant’s lives beyond HIV prevention.

### Strengths and Limitations

One limitation to this study is the risk for social desirability influencing participants’ responses during SIDIs. Qualitative interviewing staff were distinct from clinical staff to reduce social desirability, and whenever possible, the same interviewer was used for each interview to help build rapport and trust between the interviewer and participant and encourage the participant to feel free to share her honest opinions. Nevertheless, the study setting, incentives, and the relationship with staff can make participants feel obligated to speak more highly of the study products than they would otherwise. The multiple reports of challenges that participants did experience, and fact that all SIDI participants chose a product during the last six months of the study despite an option to use neither suggested a true interest in biomedical HIV prevention which aligned with reported use experiences. As described here and in other analyses of data from the REACH study [25, 26, 36], the resources and support provided to participants to support product use and adherence was extensive, and may not be feasible in real world settings. Further research that can prioritize the strategies and support mechanisms that best help AGYW use HIV prevention products as intended is warranted and will be key for successful product rollout in clinical settings with limited resources.

The random selection of serial IDI participants (with a 100% acceptance rate) from the overall REACH participant population and stratification of participants according to product use sequence and age group are strengths of this study. Another unique strength of this study was the longitudinal data collection and analysis. This allowed exploration of progressive experiences with product use, reflection on past product use compared to current product use, reflection on a decision-making process around the selected product, and non-study-related factors that shifted throughout product use and may have impacted each participants’ use of oral PrEP and the vaginal ring.

## Conclusion

In serial in-depth interviews, REACH participants described their experience and challenges they encountered to using oral PrEP and a vaginal ring for HIV prevention. Through a mix of individual-level drivers and strategies, and support through study-specific structures as well as from people in their social circles, participants were often able to overcome these challenges. The element of informed choice—being able to choose between oral PrEP or a vaginal ring—was an added mechanism that participants saw as enabling them to overcome specific barriers to product use, achieve a desired level of adherence, and reach their HIV prevention goals. In turn, being able to achieve their HIV prevention goals appeared to empower some participants to feel more agency over their broader goals for their future. Future efforts to roll-out these HIV prevention methods, along with others, should utilize a variety of support mechanisms. These support mechanisms should allow for adaptability to individual challenges and available resources while capitalizing on the ability of choice to empower PrEP users to reach their short and long-term goals for HIV prevention and beyond.

## Data Availability

Full transcripts will not be made publicly available due to the sensitive nature of the information contained therein, and the inability to ensure that a full transcript is completely de-identified. While direct identifiers and location names were not included in transcripts when they were prepared, there are no standardized procedures and tools for de-identifying sensitive qualitative data and removing indirect identifiers. In addition, qualitative research participants were assured during the informed consent process that their identity would remain confidential. Upon request, approval to access these data may be provided through a Data Use Agreement by RTI International. Data access requests may be submitted to: Mary Kate Shapley-Quinn (Project Manager, RTI International), mshapley@rti.org.
